# The impact of telemedicine on physician time invested in primary care: a large population-based descriptive study

**DOI:** 10.1017/S1463423625100364

**Published:** 2025-08-15

**Authors:** Avivit Golan-Cohen, Shlomo Vinker, Eugene Merzon, Ilan Green, Ariel Israel

**Affiliations:** 1 Leumit Health Services, Tel Aviv University, Tel Aviv, Israel; 2 Department of Family Medicine, Faculty of Medicine, Tel Aviv University, Tel Aviv, Israel; 3 Adelson School of Medicine, Ariel University, Ariel, Israel

**Keywords:** Accumulated Annual Duration of Time, primary care physicians, telemedicine, time demand, workload

## Abstract

**Aims:**

To evaluate the impact of telemedicine on the workload of primary care physicians (PCPs).

**Background:**

Telemedicine, including video visits, telephone visits, and digital correspondence, is increasingly offered by physicians, particularly since the COVID-19 pandemic. It is still unclear whether increasing the variety of services creates an increase in demand and therefore causes an increase in the workload of PCPs. In this study.

**Methods:**

A population-based descriptive study, conducted during the 2020–2021 period, on a cohort of 464,119 patients, all members of Leumit Health Services and without a diagnosis of COVID-19. The patients were stratified into three distinct groups based on the nature of their healthcare visits: Patients who used only face-to-face (FTF) visits; Patients who used asynchronous visits with or without FTF visits but did not use synchronous telemedicine visit; and patients who used synchronous telemedicine visits with or without other types of visits. We performed a comparative analysis on Accumulated Annual Duration of Time (AADT) as an index for workload, across the different periods, using standard descriptive statistics methods.

**Findings:**

Telemedicine use was higher in older persons, females, those of higher socioeconomic status, and patients with comorbidities. The greater the number of telemedicine visits, the greater the time the PCP spent on visits during the year. The largest increase in AADT (56.1%) was observed in patients who had only FTF meetings in 2020 but in 2021 made all types of visits. Overall, there was an increase of 2.5% in time invested in 2021 and a 5.8% increase in the number of patients making digital visits.

**Conclusions:**

Policymakers encouraging telemedicine should consider the additional load on PCPs associated with telemedicine use and plan resources accordingly.

## Introduction

Telemedicine provides remote clinical services to patients without an in-person visit using digital communication. It can be synchronous in audio and video or asynchronous, usually using various types of correspondence (Hollander and Carr, 2020). Until the outbreak of the COVID-19 pandemic, telemedicine was carefully implemented and under strict regulation, as it was a new technology that had to establish its pros and cons. The discerned advantages of this technology due to its paramount importance in preventing the spreading of infection resulted in its rapid assimilation while waiving some of its restrictions.

As early as 2015, US policymakers expressed concern that telemedicine would not be an alternative to face-to-face (FTF) visits but would create excess demand and increase total healthcare expenditure (Medicare Payment Advisory Commission, 2016; Uscher-Pines and Mehrotra, 2014). Data from these years revealed that, within a year of the implementation of synchronous telemedicine visits, even with an initial decrease in FTF visits, there was a return to the previous usage pattern without a significant change in the use of telephone or video meetings (Shah *et al.*, 2018). Studies that examined the influence of the implementation of correspondence with a doctor on outpatient visits reported inconsistent findings (Harris *et al.*, 2009; North *et al.*, 2014).

Patients perceived FTF visits as having a more significant contribution to health, despite the accumulation of data showing the benefits of telemedicine in various clinical situations (De Albornoz *et al.*, 2022; Downes *et al.*, 2017; Ignatowicz *et al.*, 2019). They most used telemedicine visits for administrative needs, minor medical conditions, and convenience (Hammersley *et al.*, 2019; Polinski *et al.*, 2016). It was also found that, compared with FTF visits, telemedicine visits were associated with significantly higher rates of total short-interval follow-ups every 0 to 15 days (Chavez *et al.*, 2022). These findings reinforce the concern that adding telemedicine visits will not replace FTF visits but will add to them.

Among other beneficial effects of telemedicine, it was found that it has the advantage of improving adherence to follow-up and treating patients with chronic diseases who had difficulty getting to the clinic (Barbosa *et al.*, 2020). Other studies showed that telemedicine visits reduce no-shows by 13% (Adepoju *et al.*, 2022). Increasing accessibility also raises the possibility that a greater variety of visit types will increase the total number of visits.

During the beginning of the COVID-19 pandemic, there was a significant decrease in FTF visits and a considerable increase in telemedicine visits (Patel *et al.*, 2021a; Rodriguez *et al.*, 2021). However, as early as the end of 2020, the number of FTF visits returned to a level similar to before the pandemic (Ateev Mehrotra *et al.*, 2020; Matt McGough *et al.*, 2023). At the same time, the number of telemedicine visits decreased but did not return to the low level before the pandemic. In the United States, for example, telemedicine use declined from 32% of all outpatient visits immediately after the onset of the pandemic to 13–17% of current outpatient visits. Still, this represents 38 times greater telemedicine use than before the pandemic (Oleg Bestsennyy *et al.*, 2021).

The present study aimed to evaluate the effect of telemedicine on the primary care physician (PCP) workload. International comparisons of the workload in primary care are usually made by counting the number of visits to a doctor, since this is widely reported. However, this measurement does not consider the differences in time investment, which is influenced by the patient’s condition and other personal characteristics, the treatment methods of the workplace, and the method of payment (Irving *et al.*, 2017). Counting visits to assess the load in an era where different types of visits are used could be even more misleading.

Our study used the Accumulated Annual Duration of Time (AADT) measure, which sums the minutes a patient has accumulated with a PCP during a year. This measure has previously been demonstrated by Vinker et al. as a more accurate measure to evaluate workload in primary care since it enables a comparison between different healthcare systems, even when it comes to populations with different characteristics or when the work methods are different (Nathan *et al.*, 2017).

To evaluate the effect of telemedicine on the workload, we compared AADT in 2020 and 2021 in various groups of patients in Leumit Health Services (LHS), a large health maintenance organization (HMO) that provides service all over Israel.

## Methods

### Study design: A large population-based descriptive study

Population: The population of this study consisted of all patients who were LHS members throughout the study period, had at least one consultation with a PCP in 2020 and one in 2021, and did not have confirmed COVID-19 in the calendar years 2020-2021.

LHS, one of Israel’s four HMOs, serves approximately 710,000 enrollees. Each member is primarily linked to a PCP; however, they have the flexibility to consult a different PCP should their designated one be unavailable. In 2013, LHS began offering asynchronous telemedicine consultations, expanding this with the addition of video-based medical consultations at the close of 2018. The integration of telephone consultations was expedited in early 2020 as a direct response to the unfolding epidemic. These telemedicine services were seamlessly incorporated into PCPs’ daily practices and adhered to the regulations set forth by the Israeli Ministry of Health. During this time, patient autonomy was prioritized, allowing them the freedom to select their preferred mode of consultation without restriction. In alignment with LHS regulations, eligibility for telemedicine consultations, whether synchronous or asynchronous, necessitates at least one FTF session in the preceding 12 months. The compensation structure for such consultations did not undergo any changes during the study interval, maintaining parity in the fees charged for both FTF and any form of telemedicine consultation.

The population was divided into three groups according to the utilization of the different visit modalities in 2020:

1. ***FTF group*** - Patients who used only face-to-face visits.
2. ***A-syn group*** - Patients who used asynchronous visits (correspondence with a doctor) with or without face-to-face visits but did not use synchronous telemedicine visits.
3. ***Syn group*** - Patients who used synchronous telemedicine visits (by phone or video), with or without other types of visits.

We excluded the COVID-19 patients since, for this group, the use of services was heavily influenced during the disease, due to isolation guidelines and the Ministry of Health regulations for proactive telephone follow-up.

### Study period: The 2020 and 2021 calendar years

Data: Data were retrieved from the LHS central computerized database. All the visits to the PCP were fully computerized, and the information from the electronic medical records was retrieved from a central repository. The duration of visits of LHS members to a PCP were retrieved, and the AADT was calculated for each patient. Visits shorter than one minute and video consultations that were not completed due to technical issues, were excluded from the analysis. The electronic medical record system provides accurate data on the length of each consultation. This precision is achieved as the doctor is obliged to access the patient’s electronic record at the start of any visit modality to review their personal data, medical history, and to document the specifics of the encounter. The termination of a consultation is distinctly recorded when the doctor completes their entries and closes the patient’s record, an essential action before addressing any further responsibilities.

Additional patient data included: age, gender, socioeconomic status (SES, defined on a scale of 1 to 20, as defined by the Central Bureau of Statistics in Israel according to the socioeconomic characterization of 1,629 geographical units in the state), BMI, smoking status, and the diagnosis of common chronic diseases (hypertension, diabetes, and congestive heart failure).

The study was approved by the Leumit Health Services’ Review Board and the Ethics Committee/IRB of Shamir Medical Center (ID number 149-20-LEU). All methods were performed in accordance with the relevant guidelines and regulations.

Statistical analysis: Data were extracted from the data warehouse and deidentified before analysis using Python and SQL software. Standard descriptive statistics were used to show the distribution of the data. The annual duration of visits (in minutes) was analyzed as a continuous parameter. All analyses were carried out using R statistical software.

## Results

Table 1 shows the characteristics of the study population: 52 % were female, the average age was 36.6 ± 24.4 years, and the mean SES was in the low-medium level. For the population in which the BMI was recorded (85.4%), 20.86% were obese, and most (78.4%) did not smoke (smoking status was documented in 66.3% of the overall population).

**Table 1.** Characteristics of the study population

**Characteristic**		**N (%) (unless stated otherwise)**
**N**		464,119
**Age in years**	**Mean (SD.)**	36.61 (24.37)
**Age categories**	**0–2**	17,466 (3.8 %)
	**3–9**	63,126 (13.6 %)
	**10–18**	60,264 (13.0 %)
	**19–39**	121,471 (26.2 %)
	**40–59**	99,709 (21.5 %)
	**60–79**	83,168 (17.9 %)
	**80**-	18,915 (4.1 %)
**Gender**	**Male**	223,477 (48.2 %)
	**Female**	240,629 (51.8 %)
**Socio-economic status**	**Mean (SD.)**	8.42 (3.66)
	**Missing data**	50,902 (12.32 %)
**BMI**	**Mean (SD.)**	24.85 (6.62)
	**Missing data**	67,787 (17.10 %)
**BMI categories**	**<18.5 Underweight**	76,190 (19.2 %)
	**18.5-24.9 Normal**	130,605 (33.0 %)
	**25-29.9 Overweight**	106,736 (26.9 %)
	**30 < Obese**	82,798 (20.86 %)
**Smoking Status**	**Non-smoker**	236,701 (77.0 %)
	**Past smoker**	5,540 (1.8 %)
	**Smoker**	65,330 (21.2 %)
	**Missing data**	156,548 (33.7 %)
**Chronic diseases**	**Hypertension**	115,191 (24.8 %)
	**Diabetes mellitus**	50,318 (10.9 %)
	**Congestive heart failure**	6,902 (1.5 %)
	**Ischaemic heart disease**	21,326 (4.6 %)
**2020 population**	FTF*	207,919 (44.8 %)
	A-syn group**	76,848 (16.6 %)
	Syn group***	179,352 (38.6 %)
**2021 population**	FTF*	190,594 (41.1 %)
	A-syn group**	83,691 (18.0 %)
	Syn group***	189,834 (40.9 %)

*FTF- Face To face.

**A-syn group: asynchronous remote medicine (correspondence with a doctor).

*** Syn group: synchronous remote visits (phone or video).

Table 2 describes the distribution of the above variables in 2020 and 2021 divided by the pattern of services the patients used. The data shows that in 2021 there was an 8.4% decrease in the total number of patients who performed only FTF visits; their proportion of the total population decreased by 3.7%. There was an increase of 8.9% in the total number of patients who used asynchronous visits with or without FTF visits; their proportion in the total population increased by 1.4%. The total number of patients who used synchronous visits (by phone or video), with or without other types of visits, increased by 5.8%, while their proportion in the total population increased by 2.3%.

**Table 2.** Characteristics of the study population in 2020 and 2021 by the pattern of services used

2020 Characteristics		FTF	A-syn group	Syn group
**N**		207,919	76,848	179,352
**Age in years**	**Mean (SD.)**	31.28 (22.62)	43.76 (24.30)	39.71 (25.03)
**Age categories**	**0–2**	8,825 (4.2 %)	1,248 (1.6 %)	7,393 (4.1 %)
	**3–9**	34,585 (16.6 %)	7,018 (9.1 %)	21,523 (12.0 %)
	**10–18**	35,606 (17.1 %)	7,596 (9.9 %)	17,062 (9.5 %)
	**19–39**	56,542 (27.2 %)	18,511 (24.1 %)	46,418 (25.9 %)
	**40–59**	42,761 (20.6 %)	17,875 (23.3 %)	39,073 (21.8 %)
	**60–79**	24,974 (12.0 %)	20,037 (26.1 %)	38,157 (21.3 %)
	**80**-	4,626 (2.2 %)	4,563 (5.9 %)	9,726 (5.4 %)
**Gender**	**Male**	108,407 (52.1 %)	36,154 (47.0 %)	78,916 (44.0 %)
	**Female**	99,507 (47.9 %)	40,692 (53.0 %)	100,430 (56.0 %)
**Socio-economic status**	**Mean (SD.)**	7.14 (3.27)	10.16 (3.45)	9.14 (3.67)
	**Missing data**	23,994 (11.54 %)	8,612 (11.21 %)	18,296 (10.20 %)
**BMI**	**Mean (SD.)**	24.16 (6.71)	25.62 (6.27)	25.28 (6.60)
	**Missing data**	35,306 (16.98 %)	7,862 (10.23 %)	24,619 (13.73 %)
**Smoking status**	**Non-smoker**	91,017 (74.0 %)	46,455 (79.5 %)	99,229 (78.7 %)
	**Past smoker**	1,669 (1.4 %)	1,240 (2.1 %)	2,631 (2.1 %)
	**Smoker**	30,314 (24.6 %)	10,737 (18.4 %)	24,279 (19.2 %)
	**Missing data**	84,919 (40.8 %)	18,416 (24.0 %)	53,213 (29.7 %)
**Chronic diseases**	**hypertension**	40,979 (19.7%)	24,988 (32.5 %)	49,224 (27.4 %)
	**Diabetes mellitus**	15,705 (7.6 %)	11,678 (15.3 %)	22,935 (12.9 %)
	**Heart failure**	24,451 (11.8 %)	21,235 (27.8 %)	40,396 (22.7 %)
	**Ischaemic heart disease**	5,733 (2.8 %)	5,280 (6.9 %)	10,313 (5.8 %)

2021 Characteristics		FTF	A-syn group	Syn group
**N**		190,594	83,691	189,834
**Age in years**	**Mean (SD.)**	31.82 (23.27)	41.76 (24.36)	39.14 (24.61)
**Age categories**	**0–2**	7,651 (4.0 %)	1,790 (2.1 %)	8,025 (4.2 %)
	**3–9**	33,092 (17.4 %)	8,398 (10.0 %)	21,636 (11.4 %)
	**10–18**	31,953 (16.8 %)	9,132 (10.9 %)	19,179 (10.1 %)
	**19–39**	49,567 (26.0 %)	21,118 (25.2 %)	50,786 (26.8 %)
	**40–59**	38,549 (20.2 %)	18,767 (22.4 %)	42,393 (22.3 %)
	**60–79**	24,694 (13.0 %)	20,161 (24.1 %)	38,313 (20.2 %)
	**80**-	5,088 (2.7 %)	4,325 (5.2 %)	9,502 (5.0 %)
**Gender**	**Male**	100,921 (53.0 %)	40,614 (48.5 %)	81,942 (43.2 %)
	**Female**	89,669 (47.0 %)	43,072 (51.5 %)	107,888 (56.8 %)
**Socio-economic status**	**Mean (SD.)**	7.02 (3.22)	9.99 (3.53)	9.11 (3.65)
	**Missing data**	22,242 (11.67 %)	9,643 (11.52 %)	19,017 (10.02 %)
**BMI**	**Mean (SD.)**	24.20 (6.77)	25.30 (6.24)	25.27 (6.60)
	**Missing data**	33,759 (17.71 %)	9,253 (11.06 %)	24,775 (13.05 %)
**Smoking status**	**Non-smoker**	81,624 (73.5 %)	48,641 (78.7 %)	106,436 (79.0 %)
	**Past smoker**	1,432 (1.3 %)	1,359 (2.2 %)	2,749 (2.0 %)
	**Smoker**	27,964 (25.2 %)	11,784 (19.1 %)	25,582 (19.0 %)
	**Missing data**	79,574 (41.8 %)	21,907 (26.2 %)	55,067 (29.0 %)
**Morbidity**	**Patients with hypertension**	38,199 (20.0 %)	25,679 (30.7 %)	51,313 (27.0 %)
	**Patients with Diabetes mellitus**	15,936 (8.4 %)	11,438 (13.7 %)	22,944 (12.2 %)
	**Patients with Congestive heart failure**	24,653 (13.0 %)	20,807 (25.0 %)	40,622 (21.6 %)
	**Patients with Ischaemic heart disease**	6,073 (3.2 %)	5,172 (6.2 %)	10,081 (5.4 %)

FTF- Face To face.

A-syn group: asynchronous remote medicine (correspondence with a doctor).

Syn group: synchronous remote visits (phone or video).

There is a preference for FTF visits in childhood, while in older ages (>60 years), there is greater use of digital synchronous and asynchronous visits.

Females and patients of higher socioeconomic status use more digital visits. The proportion of smokers in the non-digital group was higher. Still, digital patients suffered from almost double the rate of common chronic diseases, and their average BMI was slightly higher. All the trends remained similar in the two years sampled, and given the large data size, all differences are considered significant (*p* < 0.0001).

Table 3 describes the change that occurred in the AADT measure of visits to the PCP between 2020 and 2021 in each subpopulation relative to the characteristics of each visit.

**Table 3.** Changes in AADT between 2020 and 2021

2020	2021	count	mean AADT 2020	sd AADT 2020	mean AADT 2021	sd AADT 2021
**FTF group**	**FTF group**	146,859	23.25	28.67	24.12	29.54
	**A-syn group**	15,883	22.17	26.96	27.18	31.45
	**Syn group**	45,177	22.82	28.14	35.61	38.43
**A-syn group**	**FTF group**	9,672	31.99	35.72	27.09	33.69
	**A-syn group**	38,331	29.28	30.44	28.67	30.67
	**Syn group**	28,845	34.91	35.93	48.02	44.83
**Syn group**	**FTF**	34,063	35.22	38.15	25.87	32.35
	**A-syn group**	29,477	44.57	42.49	33.44	35.86
	**Syn group**	115,812	55.58	54.42	57.26	56.39

A-syn group: asynchronous remote medicine (correspondence with a doctor).

Syn group: synchronous remote visits (phone or video).

FTF- Face To Face.

**Figure 1.**
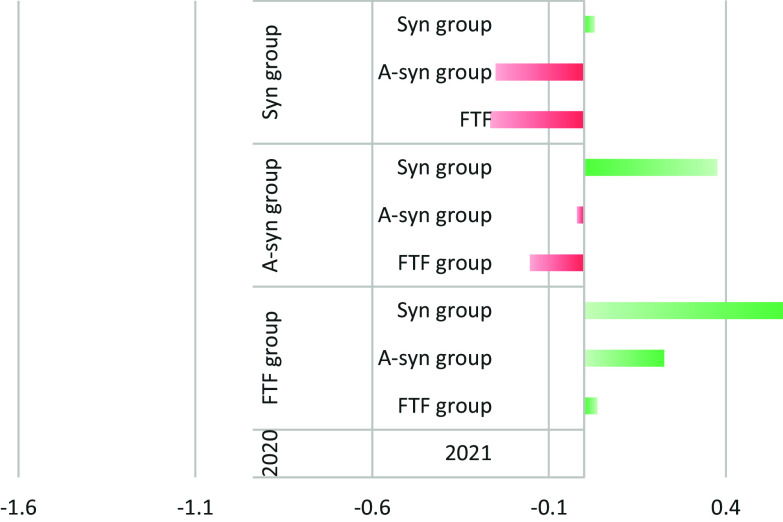
The trends of the change in AADT between 2020 and 2021. A-syn group: asynchronous remote medicine (correspondence with a doctor). Syn group: synchronous remote visits (phone or video). FTF- Face To Face.

Figure 1 demonstrates the trends of these changes.

There is an overall increase of 2.5% in the average time spent on each patient in 2021 compared to 2020. The largest increase in AADT (56.1%) was observed in patients who had only FTF meetings in 2020 but in 2021 used all types of visits. The most significant decrease in AADT (-26.5%) was in those who used all types of visits in 2020, but in 2021 used only FTF visits. For those who did not change their usage pattern between 2020 and 2021, the changes in AADT were minimal. In the other sub-groups, the greater the use of digital visits, the greater the time the PCP spent on visits during the year.

## Discussion

Limited and inconclusive data exist on the balance between telemedicine supply and demand. Some studies suggest an increase in follow-up appointments post-telemedicine visits (McGrail *et al.*, 2017; McKinstry *et al.*, 2011), while others find no difference (Shi *et al.*, 2018) or a decrease in repeat visits (Gordon *et al.*, 2017). Additionally, there are conflicting findings on the efficiency of telemedicine visits, with some reporting shorter and more effective appointments (Cravo and Hashiguchi, 2020; Downes *et al.*, 2017), while others observe expanded patient accessibility leading to an increased number of encounters (De Albornoz *et al.*, 2022; Ekeland *et al.*, 2010; Flodgren *et al.*, 2015).

In our study, we utilized the AADT index to measure the cumulative load, aiming to counteract biases and confounding factors. This approach offers a straightforward means to gauge primary time investments, facilitating informed decision-making for resource reorganization. The study encompassed 464,119 non-COVID-19 diagnosed patients from LHS over 2020-2021, categorized into three groups based on their 2020 visit patterns. We then compared the average AADT values in 2020 and 2021 for each group. By evaluating the population internally, we could mitigate confounding variables related to health and digital literacy, ultimately highlighting differences in visit utilization patterns.

Our findings revealed that older patients, individuals with higher morbidity, women, and those from higher socioeconomic backgrounds exhibited a greater propensity for engaging in telemedicine visits. Interestingly, our study contradicts previous assertions, indicating that when it comes to visits to their regular primary care physician, individuals over 60 are utilizing digital visits, sometimes even to a greater extent than younger patients, for consultations with their regular PCP. Additionally, our results corroborate prior limited data, indicating that patients with more complex health issues tend to predominantly opt for telemedicine visits with physicians they have previously seen for FTF visits (McGrail *et al.*, 2017). It is plausible that patients with greater health concerns may prefer fewer FTF visits or choose different visit types to address their diverse needs, leading to an accumulation of more treatment time over the course of a year.

Our study shows that the greater the number of digital visits, the greater the time the PCP spends on visits during the year. The most significant increase in AADT (56.1%) was observed in patients who had only FTF meetings in 2020 but in 2021 used all types of visits. The most significant decrease in AADT (-26.5%) was in those who used all kinds of telemedicine in 2020, but in 2021 used only FTF visits. These trends were also demonstrated consistently in the other groups and support the hypothesis that adding telemedicine adds to the workload imposed on the PCP.

At a system-wide scale, there was a 2.5% uptick in the total time invested during 2020 and 2021. This increase was driven by a 5.8% rise in the number of patients engaging in telemedicine visits of all types and a 1.4% surge in those opting for asynchronous telemedicine appointments, while there was an 8.9% decline in patients using only FTF visits. As a result of the COVID-19 pandemic, many PCPs can offer telemedicine. Now, when the limitation of FTF visits decreases, the question of whether the additional supply of services increases demand becomes highly relevant. The 2.5% increase in demand may translate into an additional investment in personnel resources that must be considered.

## Limitations and strengths

Our data refer only to PCPs and only to one health organization in Israel. As most telemedicine services in the ambulatory setting are currently performed in primary care, and since this is a large unselected cohort, it can be assumed that based on these findings, significant decisions can be made about the future of telemedicine services.

Although we did not include patients infected with COVID-19 in the cohort, the pandemic is characterized by individual changes in morbidity due to prolonged quarantines, fear of exposure to infection, and changes in lifestyle that were not taken into account. These changes in individual morbidity that can cause suppressed demand and additional future load (Demeke *et al.*, 2021; Patel *et al.*, 2021b) require further investigation.

In the LHS working model, all types of visits receive the same financial compensation. This financial model facilitates the study of patient and doctor behaviours while neutralizing other critical biases. Health systems with different compensation methods must consider that the financial effect was not accounted for when calculating the cumulative time investment per patient. Despite the large sample size, caution is warranted when generalizing these findings to other health contexts with different remuneration and infrastructure models, as the data were derived from a single health organization in Israel.

In this study, we did not examine whether the additional time investment is accumulated from many short return visits or the ability to invest more in each visit while reducing their total number. This question may be necessary for understanding the effect of the additional investment on the quality of care and the impact of the increased load on the burnout of the PCPs. We assume that any further investment of time will result in the worsening of burnout. Still, we must continue investigating this question and its effect on the quality of care.

This study did not address several important topics, including the effect of differences in AADT on patient health outcomes and the impact of various teamwork models on the distribution of time spent by therapists with diverse expertise in treating chronic patients. Additionally, the investigation of predominant types of demands in digital consultations was not included. Expanding future research to cover these areas will provide a deeper understanding of the issues that need to be addressed when planning interventions aimed at balancing demands.

## Conclusions

Patients using telemedicine create an additional burden on the PCP. For the most part, these are older, sicker patients, from a higher socioeconomic status, for which it is possible that the expansion of the services offered, corresponds to the variety of their needs. As of today, this is an increase of a few percentages, but in view of the whole system and assuming that this is an initial manifestation of the phenomenon, the policymakers must take into account the organizational change that will be required to deal with it.

## Data Availability

The datasets analyzed during the current study are not publicly available because it is business information but are available from the corresponding author upon reasonable request.

## List of abbreviations

AADT- Accumulated Annual Duration of Time

PCP- Primary care physician

LHS- Leumit Health Services

HMO- Health Maintenance Organization

FTF- Face to Face
